# Piezoelectric biosensors: shedding light on principles and applications

**DOI:** 10.1007/s00604-024-06257-9

**Published:** 2024-03-07

**Authors:** Petr Skládal

**Affiliations:** Department of Biochemistry, Faculty of Science, Masaryk University, Kamenice 5, 62500 Brno, Czech Republic

**Keywords:** Quartz crystal microbalance, Immunosensors, Enzyme activity, Cellular biosensors, Microbial detection, Combined biosensing set-ups

## Abstract

The three decades of experience with piezoelectric devices applied in the field of bioanalytical chemistry are shared. After introduction to principles and suitable measuring approaches, active and passive methods based on oscillators and impedance analysis, respectively, the focus is directed towards biosensing approaches. Immunosensing examples are provided, followed by other affinity sensing approaches based on hybridization of nucleic acids, aptamers, monitoring of enzyme activities, and detection of pathogenic microbes. The combination of piezosensors with cell lines and testing of drugs is highlighted, including mechanically active cells. The combination of piezosensors with other measuring techniques providing original hybrid devices is briefly discussed.

**Graphical Abstract:**

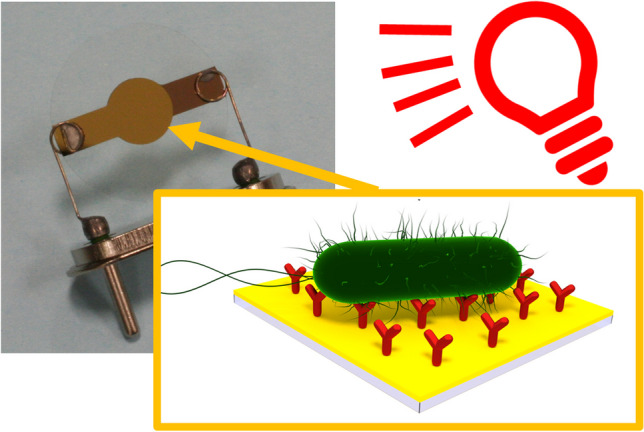

## Introduction

Compared to traditional approaches that rely on labels such as enzymes, fluorophores, and radioactivity, piezoelectric sensors provide a direct method for real-time monitoring of biointeractions at the sensor surface, resulting in simplified assay formats. Continuously evaluating the progress of the interaction provides more detailed information than traditional techniques that measure only the amount of label bound at the end of the binding process. Direct affinity sensors can be used repeatedly in a wide range of measurements, which has the advantage of lower cost per one assay. Changes in surface mass resulting from the formation of affinity complexes at the sensitive site can be measured by a variety of physical transducers. Advanced optical systems are most commonly used. However, piezoelectric (PZ) devices offer a similar but significantly less expensive alternative [1].

**Fig. 1:**
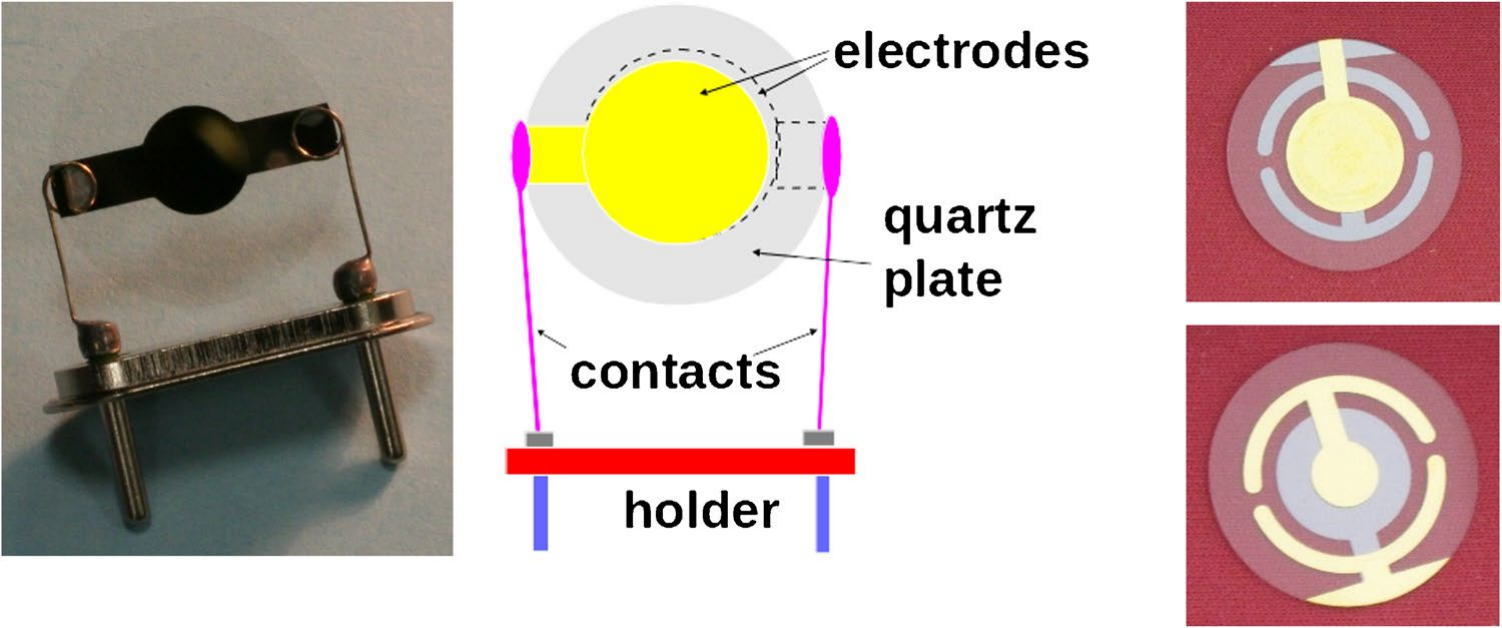
Piezoelectric quartz crystal resonators. The typical 10 MHz PZ crystal with gold electrodes mounted on a holder (left), Schema of this design (middle). Alternative arrangement of gold electrodes providing contacting pads on the same side for simplified use in cells for the use in liquid (right)

Several anisotropic crystals exhibit the piezoelectric effect. This means that mechanical deformation of these crystals produces oriented dipoles and electrical voltage. Conversely, an alternating voltage applied to these crystals excites vibrations. At resonance, when the frequency equals the natural vibration frequency, energy transfer from the electric field to the crystal is most efficient, and energy remains in the vibrating system. Several types of PZ and acoustic transducers are available, but this text will focus only on the classic quartz crystal microbalance (QCM). As shown in Fig. 1, QCMs consist of quartz plates coated on both sides with metallic electrodes. The vibrations are known as the thickness shear mode or the bulk acoustic mode, and they occur in the area where the electrodes overlap.

Figure 1 shows the typical piezoelectric quartz crystals along with their description. A thin plate of quartz (AT-cut) is coated with metal electrodes on both sides. Gold is deposited over a thin chromium interlayer to serve as an inert surface for biomolecular and cellular applications. The plate is conveniently inserted in a holder for simplified manipulation and connection to electronics. Piezoelectric transducers have been utilized as chemical sensors ever since Sauerbrey discovered the relationship between the mass of adsorbed films and the resonant frequency [2]:

1 $$\Delta f=-\frac{2{f}_{0}^{2}\Delta m}{A{\left({\rho }_{q}{\mu }_{q}\right)}^{1/2}}=-2.26\cdot {10}^{-6}{f}_{0}^{2}\frac{\Delta m}{A}$$

The change Δ*f* of the resonant frequency *f*_0_ of piezocrystals is proportional to the mass change Δ*m*, the other parameters represent* A* active area of the resonator, which is given by the overlapped electrodes, and parameters of the quartz—density ρ_q_ and shear modulus μ_q_, respectively. Alternatively, the simplified form with numeric constant applies to calculations using Δ*f* in Hz, *f*_0_ in MHz, and Δ*m* in g/cm^2^. The typical working frequencies are from 5 to 20 MHz; higher values are not used as the quartz plates become too thin. Thus, the frequency change of 1 Hz corresponds to some 17.7 and 4.4 ng/cm^2^ for the common 5 and 10 MHz PZ crystals, respectively. The system functions as a sensitive quartz crystal microbalance (QCM). In this way, the number of molecules bound on the sensitive area of electrodes can be easily quantitatively measured as a decrease of the resonant frequency. However, Eq. (1) is strictly valid for oscillation in air and only applies to rigid layers attached to the surface (e.g., electrolytically deposited metal layers).

Note that in this paper, I am trying to avoid the “microbalance” and use only piezoelectric. This is because Eq. (1) cannot be simply used in the case of viscoelastic biolayers in solution. Well, there is also a cumbersome option to detect biomolecules on the surface after drying the sensor.

It has been shown that quartz crystal microbalance measurements can be performed in liquid, in which case accompanying viscoelastic properties of the liquid medium and deposited layers related decrease in the resonant frequency will be obtained [3]:

2 $$\Delta f=-{f}_{0}^{3/2}{\left(\frac{{\eta }_{l}{\rho }_{l}}{\pi {\rho }_{q}{\mu }_{q}}\right)}^{1/2}$$

Here, *ρ*_l_ and *η*_l_ represent density and viscosity of the solution. Even though according to the recent studies the microbalance response is more complex [4], the resonant frequency serves as a convenient signal reflecting the surface-bound molecules.

Note that it might be interesting to mention also an equation providing penetration depth δ:

3 $$\delta ={\left(\frac{{\eta }_{l}}{\pi {{f}_{0}\rho }_{l}}\right)}^{1/2}$$

In water, this provides around 250 and 180 nm for 5 and 10 MHz PZ crystals, respectively. Piezoelectric systems were once overlooked or underestimated by the biosensor community due to a common belief that they were less sensitive than other types of transducers. However, as theoretical backgrounds for the operation of piezosensors in liquids became more complete and many successful applications were reported in the literature, this perception changed.

Note that in special situations, flexible spatial organization on the PZ surface can reveal anti-Sauerbrey behavior due to their variable interfacial architecture [5]. This was demonstrated on compounds with branched and flexible structures providing different viscoelastic characteristics—self-assembled monolayer consisting of tripod-like hydroxamic thioligands. The expected mass loading was accompanied with viscous damping (anti-Sauerbrey one) at the same time.

## Measuring set-ups for piezosensors

**Fig. 2:**
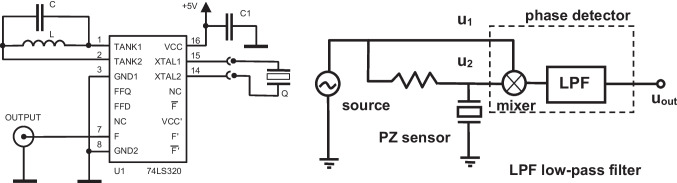
Example of the oscillating circuits for PZ crystals (Q) in solutions. The integrated digital gate oscillator based on the 74LS320 (left, based on [6]). Parts of the oscillator include C1, capacitor 100 nF; the capacitor C and inductor L depend on the resonance frequency of the chosen PZ crystal, e.g., for 10 MHz crystal, the values should be close to 17 pF and 15 µH, respectively. The resonance condition (2πf_0_)^−2^ = LC should be satisfied. Concept of the novel PZ detector based on the phase shift measurements (right, based on [7]). The PZ sensor is supplied with a constant frequency within its resonant frequency band (source); the phase shifts resulting from the PZ surface interactions are then monitored using the circuit consisting of the highly stable source of interrogation voltage and mixer-based phase shift detector

Piezoelectric crystals with gold electrodes with the basic resonant frequency of 5 to some 20 MHz are typically used. The crystals with higher frequencies will provide a higher sensitivity (Eq. 1), though the noise levels will go up and very thin quartz plates become fragile. For driving the PZ resonator, the active method is most used; the crystal becomes part of an oscillating circuit driver, and the output frequency is measured. The simplest construction is based on the gate oscillator using the integrated circuit 74LS01; the integrated form of this oscillator, 74LS320, will provide much higher energy to the crystal (Fig. 2, left) resulting in improved performance under variable conditions [6].

A frequency counter is a commonly used device in electronics for measuring changes in resonant frequency. The resolution of the frequency should be at least 1 Hz within a 1-s clock tick interval, and the upper limit of frequency should be around 50 MHz. It is important to note that the device should have the capability to connect to a personal computer for online monitoring of interactions. The easiest way was through the standard serial port (RS 232C).

To improve sensitivity, a low difference frequency signal (typically around 10 kHz) is obtained by subtracting the output frequency of the oscillator with a stable reference frequency. This signal can then be further processed using a precise frequency-to-voltage converter, filter, and fast, high-resolution A/D converter to digitize it. The direct counting might be substantially improved using the advanced beat-based procedure. Alternatively, the indirect counting approach can be chosen in which the lower experimental frequency is used as a clock signal for counting much higher stable frequency (tenths of megahertz). Such options provide shorter measuring times (1 ms) and resolutions better than 0.1 Hz. A novel method based on phase shift at a fixed frequency near the resonance was developed (Fig. 2 right) [7]; this allowed to improve the signal/noise ratio 3 times even for the common 10 MHz resonators. Evaluation of this concept using the PZ immunosensor for the pesticide carbaryl provided limits of detection (LOD) for the phase shift method 11 vs. 0.14 ng/ml when using 9 and 100 MHz resonators, respectively [8]. The relevant sensors and instruments are available (https://www.awsensors.com).

As mentioned above, the simple interpretation of resonant frequency changes according to Eq. 1 fails in case of thick viscoelastic layers adhering to the PZ sensing surface. This is a typical situation for biolayers consisting of biopolymers or cells. To provide a better understanding of such situations, the QCM-D concept and instrument was introduced by QSense (https://www.biolinscientific.com), and the common *f* is supplemented with dissipation *D* signal. The original concept is based on pinging the PZ crystal repeatedly [9]. The common PZ resonator is rapidly excited to resonance, and the driving voltage is switched off. The decay curve of the oscillation is monitored and numerically fitted to an exponentially damped sinusoid. This provides *D* along with the resonant frequency *f*. The procedure is quite fast, and the acquisition rate allows to record several higher harmonics. This indicates validity of the Sauerbrey equation; the rigid ad-layer provides Δ*fn*/*n* constant for *n*-th harmonics (overtones) and allows to investigate details of homogeneous viscoelastic films (polymers, biomolecular layers), cells and biological assemblies, particles and even conformational changes (www.biologic.net, www.biolinscientific.com). This approach is especially welcome by biologists who prefer to use complete and straightforward user-friendly instruments and do not enjoy that much the complex technical issues.

Note that the information on energy losses due to the dissipation can be easily obtained from analog lever oscillators, where the actual losses on the PZ crystal are compensated using the autogain control loop; voltage from this loop is proportional to the resistance *R* of the PZ crystal and can be recorded together with the resonance frequency shifts.

**Fig. 3:**
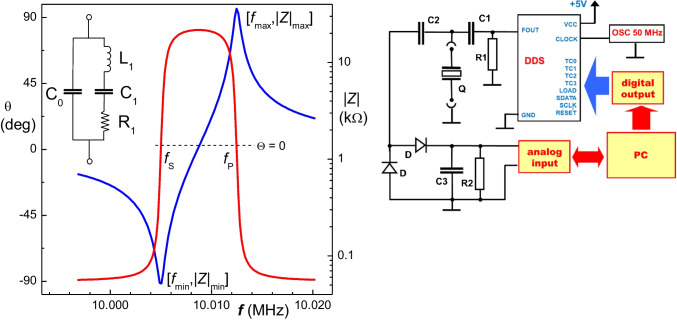
Passive method for measurements with PZ sensors using impedance spectroscopy. The impedance *Z* is represented by amplitude |*Z*| and phase shift θ plotted against the frequency near the resonance region (left). The inserted scheme represents the Butterworth-Van-Dyke equivalent circuit. The simple laboratory-made impedance analyzer suitable for monitoring the amplitude of impedance (right)

In addition to the active measuring methods for PZ crystals, the passive approach based on the impedance spectroscopy can be used [10]. The typical spectrum for the 10 MHz PZ crystal in dry state is presented in Fig. 3.

The impedance *Z* as a vector is represented by its amplitude |*Z*| and phase shift angle *θ*. The resonance defined as minimum of the impedance is found at [*f*_min_, |*Z*|_min_], followed by the antiresonance existing at [*f*_max_, |*Z*|_max_]. When the PZ crystal is part of the oscillator circuit, this becomes tuned at either *f*_s_ or *f*_p_—serial and parallel resonances, respectively, depending on construction of the circuit. The *f*_s_ and *f*_p_ values are found at the points where the phase shift *θ* is equal to zero. The values of *f*_min_ and *f*_s_ converge when the dissipation is close to zero and the same applies to *f*_max_ and *f*_p_. Consequently, when operating in gas phase, there are only two resonant frequencies.

The Fig. 3 at left also presents the Butterworth-Van-Dyke equivalent circuit approximating the given PZ resonator. It consists of several components such as capacitance C1, inductance L1, and resistance R1, in parallel with a shunt capacitance C0 (Fig. 3).

Note that the value of *L*_1_ represents the oscillating mass adhered on the PZ surface; *C*_1_ corresponds to the elasticity of the adhered layer. The *R*_1_ resistance represents the energy losses in the system, and the shunt capacitance *C*_0_ is due to the overlap of the electrodes on the crystal. The values of these components can be numerically fitted from the impedance spectrum and provide the dissipation:$D={R}_{1}/\left(2\pi f{L}_{1}\right)$

The dissipation can be also obtained from impedance minimum peak half bandwidth divided by the *f*_min_ value.

More advanced systems can employ the Mason equivalent circuit [11]. Using the boundary conditions, the Mason model succeeds in providing a more generalized response of PZ resonators coated with various types of films expanding to the surrounding liquid phase. It also helped with explanation of positive frequency changes in binding interactions.

The impedance analyzers operating in tenths of MHz region used to be large (and expensive) instruments. However, the fast progress in the RF integrated circuits and DDS synthesizers allows to construct small devices (Fig. 3, right). To complete this technical part, one should mention the OpenQCM concepts (https://www.openqcm.com) providing easily accessible and miniature PZ detectors based on the Arduino microcontroller platform well-known to hobbyists. The very compact instrument is produced by the popular 3D printing process and perhaps opens a way to “home” piezoelectric biosensing compatible with the “point of-care” trends. Now, they also provide palm-sized impedance (or network) analyzer which interrogates the PZ sensor by sweeping around its resonance frequency. The variable input frequency is generated by the AD9851 DDS/DAC frequency synthesizer, and the resultant output signal from the PZ sensor is evaluated by the AD8302 gain and phase detector. This device provides the impedance spectrum as the gain, magnitude ratio across the quartz crystal, and the phase difference between the actuation and output signals.

Simply, active measuring with oscillator is suitable for fast processes as it provides sampling intervals below 1 s; the impedance spectroscopy is generally 10 times slower but provides more details for interpretation of complex processes.

## Affinity-based bioassays

### Immunosensing

Modification of the sensing area with biorecognition molecules provides affinity biosensors. When the immobilized ligand is either an antigen (hapten) or antibody, the resulting device functions as an immunosensor specific for the corresponding binding partner. Bioligands can be immobilized using a variety of strategies, from simple physical adsorption to covalent binding to activating sublayers, and finally to oriented attachment of antibodies. The well-known immobilization procedures [12] are suitable also for piezosensors.

Note that As PZ sensors contain gold-based electrodes, the self-assembled monolayers of aminothiols and mercaptocarboxylic acids are usually deposited as the first layer. The former substances can be activated with glutaraldehyde, the latter ones using the carbodiimide/N-hydroxysuccinimide-based procedures.

The PZ immunosensors were successfully used for measuring concentration of many different analytes [13, 14]. Specialized review discussed opportunities of PZ devices in the field of novel peptide and protein-based pharmaceutical products [15]. The working formats include direct, competitive, and displacement assays. Large analytes (proteins, viruses, and bacteria) can be measured directly after binding to the immobilized capture antibody. To improve the analytical parameters, the binding of Au nanoparticles (NP) can be implemented, optionally followed by enlarging deposition of silver or gold. In case of the prostate specific antigen, such procedure improved the LOD from 690 to 50 pg mL^−1^ [16]. Tumor necrosis factor alpha (TNF alpha) is an important marker of inflammatory processes; it was directly captured on the antibody-modified PZ surface [17]. However, to enhance the sensitivity, the same antibody was linked to magnetic particles which provided substantially higher signals; the achieved LOD was 1.6 pg mL^−1^.

To achieve a measurable change in frequency for small molecules acting as haptens, a competitive measurement is necessary. The analyte is mixed with an antibody to form immunocomplexes, and the remaining free binding sites of the antibody can then interact with the sensing surface modified with a derivative of the analyte. For example, detection of sulfathiazole in honey employed the competitive format with monoclonal antibody, reaching a LOD of 100 ng kg^−1^, 50 times better compared to alternative antibiotic detection methods [18]. However, numerous papers reported that small molecules can be measured directly.

PZ immunosensing of exosomes expressing the transmembrane protein CD63 was carried out directly with immobilized capture antibody and the QCM-D system [19]. The LODs were 2.9·10^8^ and 1.4 ·10^8^ of the exosome-sized particles per mL for frequency and dissipation signals, respectively; such levels are within the physiological limits. The metastatic breast cancer is related to expression of notch-4 receptor; its antibody attached on PZ surface with poly(2-hydroxyethyl methacrylate-PHEMA) NPs allowed to detect human metastatic breast cancer cells MDA MB 231, overexpressing notch-4 on their membranes [20]. The achieved LOD was 50 cells per mL.

Surprisingly, immunoassays in organic phase are feasible. Sulfur mustard (SM, bis(2-chloroethyl) sulfide) belongs to chemical warfare agents regulated under the Chemical Weapons Convention. SM was used during the World War I and the Iran-Iraq war; it is considered as a potential chemical agent for terrorist attacks. SM is a strong alkylating agent; this was employed for simple conjugation of SM as hapten with albumin, providing 10.6 SM substituents per one molecule of albumin [21]. In reality, it is not practical to analyze sulfur mustard in water-based samples due to its rapid hydrolysis to thiodiglycol. Therefore, SM should be initially recovered by extraction using organic solvents, evaporation of the solvent follows, and SM is transferred to aqueous phase for immunoassay. For this reason, we used immunoassay in dry toluene as the carrier phase and employed direct binding of SM to the immobilized capture antibody.

### Aptamers and molecular imprinted polymers

Medical diagnostic approaches based on PZ sensor modified with aptamers were reviewed [22]; details of the SELEX procedures for optimizing aptamers for bacterial pathogens were summarized, too [23]. The aptamers interact with the analyte but also allow to involve strategies relying on multiple interactions of several nucleic acid chains containing complementary regions; this helps to enhance the resulting signals.

The thrombin biosensor consisted of an array of gold nanocages loaded with signaling molecules in their cavities and the DNA probes immobilized on their surface for hybridization with thrombin-specific aptamers to seal their pores [24]. In the presence of thrombin, the aptamers were lifted off the gold nanocages, the cargo molecules of polyamidoamine were released, and this event was followed by the PZ sensor. The signal can be amplified by the choice of cargo molecules. The LOD was 1.2 nM in human serum. A dual aptamer sandwich-like formation with target matrix metalloproteinase 9 achieved the LOD of 6.8 pM in serum [25]. When measuring small molecules using immobilized capture aptamers, some amplification strategy might be useful—Au NPs enhanced response in case of okadaic acid [26].

Molecularly imprinted polymers (MIPs) have gained significant attention in the field of (bio)sensors due to their unique features that distinguish them from natural antibodies. MIPs act as artificial antibodies and possess robustness, multiple binding sites, low cost, facile preparation, and high stability under extreme operating conditions (e.g., higher pH and temperature values).The sensitivity of a PZ sensor can be enhanced by using MIPs as synthetic receptors. This is achieved through the creation of multiplexed binding sites based on the MIP process, which utilize size, 3D-shape, and chemical function to create molecular sites complementary to the target. These memory sites are then used to capture and detect the target compound [27]. For glucose monitoring in saliva, boronate complexes were employed [28]. The microgel networks deposited on PZ sensors provided boric acid binding sites to interact with glucose. The amino acid layer wrapped around the microgel and crosslinking layer can effectively eliminate the impact of non-specific salivary proteins.

The interaction between surface bound TiO_2_ NPs and phosphate was utilized for determination of the phosphoprotein casein in milk and egg white [29]. The aminated TiO_2_ was linked to the monolayer of mercaptoundecanoic acid; the assay was completed in 5 min, and the LOD of 5.3 g µmL^−1^ was achieved.

### Detection of microbes

Continuous monitoring of pathogens finds applications in environmental, medical, and food industry settings [30], the early detection of infectious diseases being perhaps most important [31]. The SPR and PZ biosensors were compared for immunoassays of *Bacillus atrophaeus* spores. The quality of spores was significantly influenced by the cultivation, with longer sporulation periods providing better quality spores. The response of the immunosensors was also affected by the adopted immobilization strategy. The reduced antibodies have the potential to lower the limit of detection because these thiol-containing fragments bind directly to the gold surface. In this case, both SPR and PZ biosensors provided comparable results [32].

A combined review addressed PZ biosensors for detection of viruses including COVID-19 and discussed NPs (Au, Ag, Cu, Se, ZnO, MgO, carbon dots, and quantum dots) potentially useful in the antiviral therapy [33]. Another review focused on PZ biosensors for COVID-19 appeared [34].

Note that for detection of bacteria, enhanced sensitivity can be achieved using magnetic particles for preconcentration; *Campylobacter jejuni* was thus detected from poultry products from 20 CFU mL^−1^ [35]. Another option is a short precultivation time; 2 h at 37 °C helped to detect 10 mL^−1^ of *Salmonella* Typhimurium in chicken meat [36].

### Nucleic acid sensing

The detection procedures involve hybridization steps between the immobilized oligonucleotide and the complementary sequence present in sample. In case of low concentrations, amplification strategies can be applied before the PZ measurements. The direct PZ detection of non-amplified target sequence was reported, too [37]. The fragmentation of the genomic sequence with restrictase, thermal denaturation, and blocking of renaturation using short oligonucleotides was critical for successful performance.

The detection of micro-RNA molecules is often challenging; for miR-21, a sandwich hybridization between target and specially designed probes was used [38]. The procedure further employed the attachment of TiO_2_ NPs and subsequent photocatalytic silver enhancement reaction resulted in a LOD of 0.87 pM. Another approach formed miR-21 heteroduplex with complementary DNA, which subsequently intercalated the PZ-surface attached pyrene moieties, and finally, enhancement with Au NPs was realized [39]. The LOD was 3.6 pM, and miR-21 from cell culture and brain tissue was successfully determined. The interesting DNA-liposome enhancement was due to hydrodynamic reflection: a stress on the liposome surface in flow conditions propagates back to the resonator, which become sensitive to the distribution of the liposome-wall distance influenced by the mechanical properties of double-stranded-DNA chains [40].

Microbial sensing can be focused also on DNAs and RNAs present in viruses and bacteria. The RNA from the hepatitis C virus from serum was amplified using reverse transcriptase-polymerase chain reaction (RT-PCR) and the amplicons can be quickly detected using four different oligonucleotide capture probes attached to the PZ surface [41]. The hybridization was completed within 5 min and sensing surfaces were quickly regenerated using 1 mM HCl. Besides PCR, the loop-mediated isothermal amplification (LAMP) products can be bound on the PZ surface in situ; *Listeria monocytogenes* DNA served to demonstrate this real-time approach [42].

### Enzymatic activities

The classic option is monitoring of hydrogen peroxide coming from oxidase reactions. In the presence of peroxidase, e.g., chloronaphthol, insoluble product sediments on the PZ surface making a nice mass change [43].

Note that another common approach is modification of the PZ surface with substrate for the target enzymes from the hydrolases class; mass change results after cleavage of the substrate.

However, quite sophisticated approaches can be designed to measure fewer common activities. Poly(ADP-ribose) polymerase-1 (PARP-1) is highly interesting as a potential biomarker and therapeutic target for cancer. The detection of PARP-1 is necessary for early diagnosis of cancer and drug development [44]. After activation by specific DNA, PARP-1 cleaves NAD^+^ into nicotinamide and ADP-ribose to synthesize a hyperbranched poly(ADP-ribose) polymer. This negatively charged product formed a complex with positively charged cetyltrimethylammonium bromide coated on Au nanorods to enhance the response; the resulting LOD for PARP-1 was 0.04 nM.

The clinically important blood coagulation assay is a cascade of enzyme reactions and thus belongs to this part. The final product fibrin deposited on the PZ surface coated with parylene-C and the dissipation change was monitored using a custom-made system providing data to smartphone via Bluetooth wireless communication [45]. The first order derivative of the dissipation signal was used to determine the activated partial thromboplastin time and prothrombin time parameters.

For the assay of collagenase-IV, a special peptide (P1) contained a target hydrolyzed sequence (Pro-Gly), terminal Cys residue for PZ surface binding via the Au-thiol strong bond, and eventually Au NP as a heavy label to be released after the enzyme cleavage [46]. Thus, achieved LOD 1 ng mL^−1^ was 7 times improved compared to the alternative without Au NPs.

### Characterization of affinity kinetics

Affinity interactions, such as the formation of biocomplexes between antigens and antibodies, proteins and nucleic acids, and the hybridization of complementary strands of ssDNA, as well as chemoreceptor-ligand interactions, represent important biochemical processes in nature. Therefore, significant effort is devoted to the detailed study and quantitative characterization of the underlying binding reactions. Real-time monitoring of bioaffinity interactions can be achieved through direct methods that employ simplified assay formats. These methods provide more detailed information compared to traditional label-based techniques, which only measure the amount of bound label at the end of the binding process. Additionally, direct affinity sensors can be used repeatedly for many assays. Several physical transducers can measure surface mass changes resulting from the formation of biocomplexes at the sensitive area. While advanced optical systems are mostly used, piezoelectric devices are a less expensive alternative.

Note that well, as the piezosensors cannot be easily miniaturized, large flow cells require large amounts of reagents and samples.

**Fig. 4:**
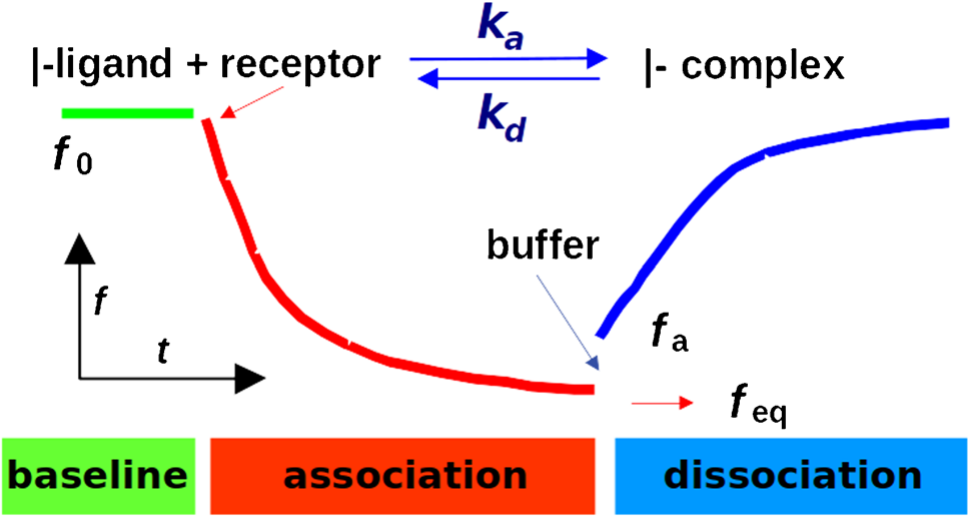
Characterization of kinetic properties of affinity interactions. First, the baseline signal is recorded using the PZ sensor with immobilized ligand (A) in the carrier buffer only (*f*_0_). The association reaction follows in the presence of the sample containing complementary receptor (B)—formation of affinity complexes (AB) at the sensing surface, resulting eventually in equilibrium *f*_eq_. A spontaneous dissociation of the complexes can start from the *f*_a_ level (equilibrium is not required)

Formation of the complex AB at the surface of the piezoelectric crystal is characterized by the kinetic association *k*_a_ and dissociation *k*_d_ rate constants (Fig. 4). The rate of formation of the complex d[AB]/dt can be expressed using the measured frequency *f* and concentration *c* for the free partner B:

4 $$\frac{d\left[AB\right]}{dt}\approx -\frac{df}{dt}={k}_{a}\left({f}_{max}-f\right)c-{k}_{d}f$$

The binding curves (*f* vs. *t* dependencies) were transformed to obtain d*f*/d*t* vs. *f* plots that subsequently provided kinetic constants using linear regression [47]; this approach becomes obsolete as non-linear regression is available on common PCs. Thus, a more elegant and precise method is integration of Eq. 4 and then introduction of substitutions *f*_eq_ and *k*_obs_ [48]. The dependence of the resonant frequency *f* on time *t* can be fitted to the kinetic equation similarly as described for the optical biosensor system [49]:

5 $$f=\frac{{k}_{a}c{f}_{max}}{{k}_{a}c+{k}_{d}}\left\{1-exp\left[-\left({k}_{a}c+{k}_{d}\right)t\right]\right\}={f}_{eq}\left[1-exp\left({k}_{obs}t\right)\right]$$

**Fig. 5:**
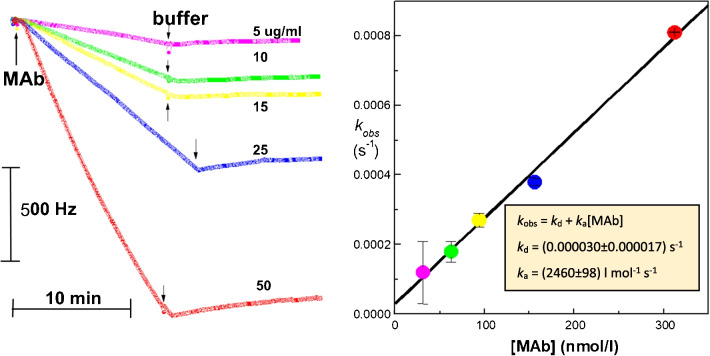
Kinetic analysis of interaction between the immobilized wheat proteins secalins and the corresponding monoclonal antibody (MAb) in solution. Binding interaction (relative frequency changes, *f*_0_ was 10 MHz)—association and dissociation traces for different MAb concentrations injected at the beginning, the arrows indicate switching to buffer flow and beginning of dissociations (left). For each association, the k_obs_ value was determined using the non-linear regression. Thus, obtained *k*_obs_ were plotted against the corresponding molar concentrations of MAb (M_r_ of antibody was assumed to be 160 kDa) and the linear fit provided the *k*_a_ and *k*_d_ values (right)

*c* is concentration of the free partner B, and *f*_max_ represents the capacity of the crystal (maximum change of frequency obtained after saturation of all binding sites). In this way, the binding curves can be used directly for calculation of parameters using non-linear regression. Plot of *k*_obs_ against (molar) concentration *c* provides values of the rate constants [50]. A typical affinity experiment with the piezoelectric immunosensor is schematically shown in Fig. 5.

The technique described above was used to characterize several monoclonal [51] as well as recombinant [50] antibodies.

Note that when dealing with polyclonal antibodies, it is not possible to obtain exact kinetic parameters. However, it is still possible to make a quantitative comparison between different products. Additionally, the fast determination of affinities is useful during the screening and development of antibodies. These kinetic parameters can help in selecting the most suitable antibody for the intended purpose when multiple alternatives are available.

This approach is not limited to immunointeractions; any affinity binding can be characterized. The extracellular vesicle populations (exomeres < 50 nm and exosomes 50–80 nm sized) interacted with four MAbs against tetraspanins (anti-CD9, anti-CD63, and anti-CD81) and recombinant intercellular adhesion protein CD54 protein [52]. Adaptive interaction distribution algorithm was designed for the analysis of the degree of heterogeneity in rate constant distributions; it demonstrated two interaction sites with comparable binding kinetics and estimated equilibrium dissociation constants *K*_d_ ranging from nM to fM. The casein-modified PZ sensors were used to study the kinetics of proteolysis with trypsin [53].

## Cell-based studies

**Fig. 6:**
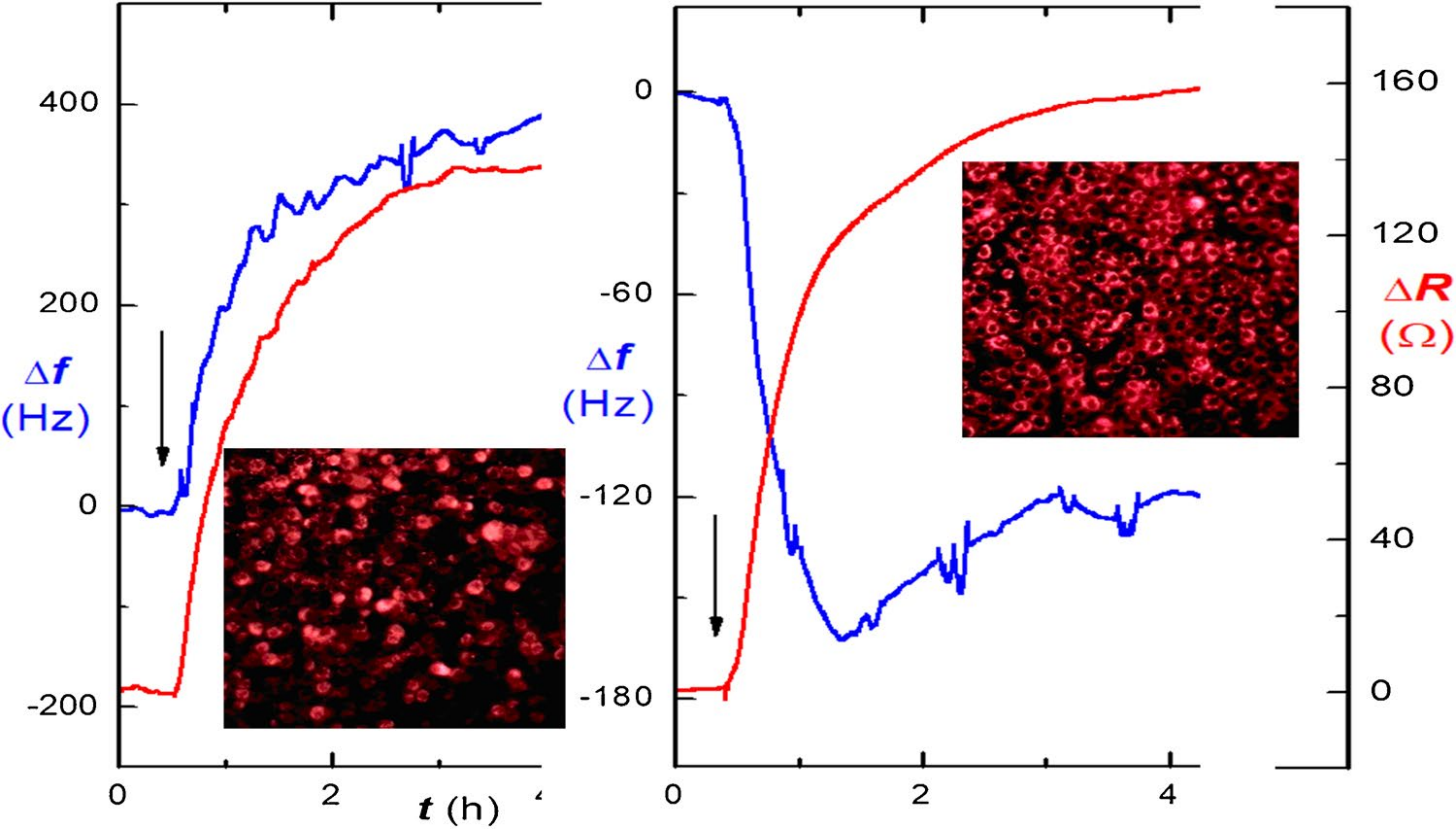
Frequency *f* and resistance *R* changes during cultivation of the lung melanoma B6F10 cells on PZ sensors modified using either laminin (left) or vitronectin (right). The arrows mark the seeding of cells on the surface. The inserted fluorescence images present the surfaces with cells labeled with the fluorescent dye specific for mitochondria MitoTracker Red CMXRos. Modified data from [55]

The various cells are easily combined with the robust and affordable PZ sensors; this area is rapidly expanding thanks to information on interfacial processes provided by the dissipation signals. Cell adhesion is an essential aspect of cellular behavior. Finding innovative methods to probe the adhesion of cells in their native state can greatly advance the understanding of control and regulation of cellular behavior and their impact on human health [54]. We tested different cell lines adherence to different surface conditions [55]; the extracellular matrix proteins vitronectin and laminin served as modifiers promoting the adhesion as shown in Fig. 6.

The lever oscillator provided changes of frequency and resistance of the modified PZ crystal; the resistance provides similar information on viscoelastic processes as dissipation. One can see quite different frequency responses of the same cells depending on the present contact proteins; this is also confirmed by fluorescence images; at left, there are many cells of circular shape indicating suboptimal adhesion and beginning of apoptosis. The increase of frequency can be at least partially attributed to the release of laminin from the surface during contact with the B6F10 cells. Actually, a similar trend was also observed for the combination of surface with laminin interacting with the WB F344 cell line; these cells completely desorbed from the surface and finished in apoptosis [55].

The suspension cells which do not adhere to the PZ surface can be attached using the glue-like layer of poly(dopamine) [56]. Such immobilization was favorable for specific interactions of cell surface glycoligands with lectins. The wireless QCM system was adopted for studies of interactions of microtubule components [57]. A recent review focused on other applications of PZ sensors for cytoskeleton biomechanics highlighted studies of cytoskeleton constituent parts and its macroscopic structure, in vitro motility events, interactions with artificial surfaces and with other cell components, such as lipid membranes or specific proteins [58]. The approach provides for noninvasive real-time recording reorganization of the cytoskeleton in situ within cells. Further, pharmaceutical investigations help understanding how cells respond mechanically to the presence of tested agents.

**Fig. 7:**
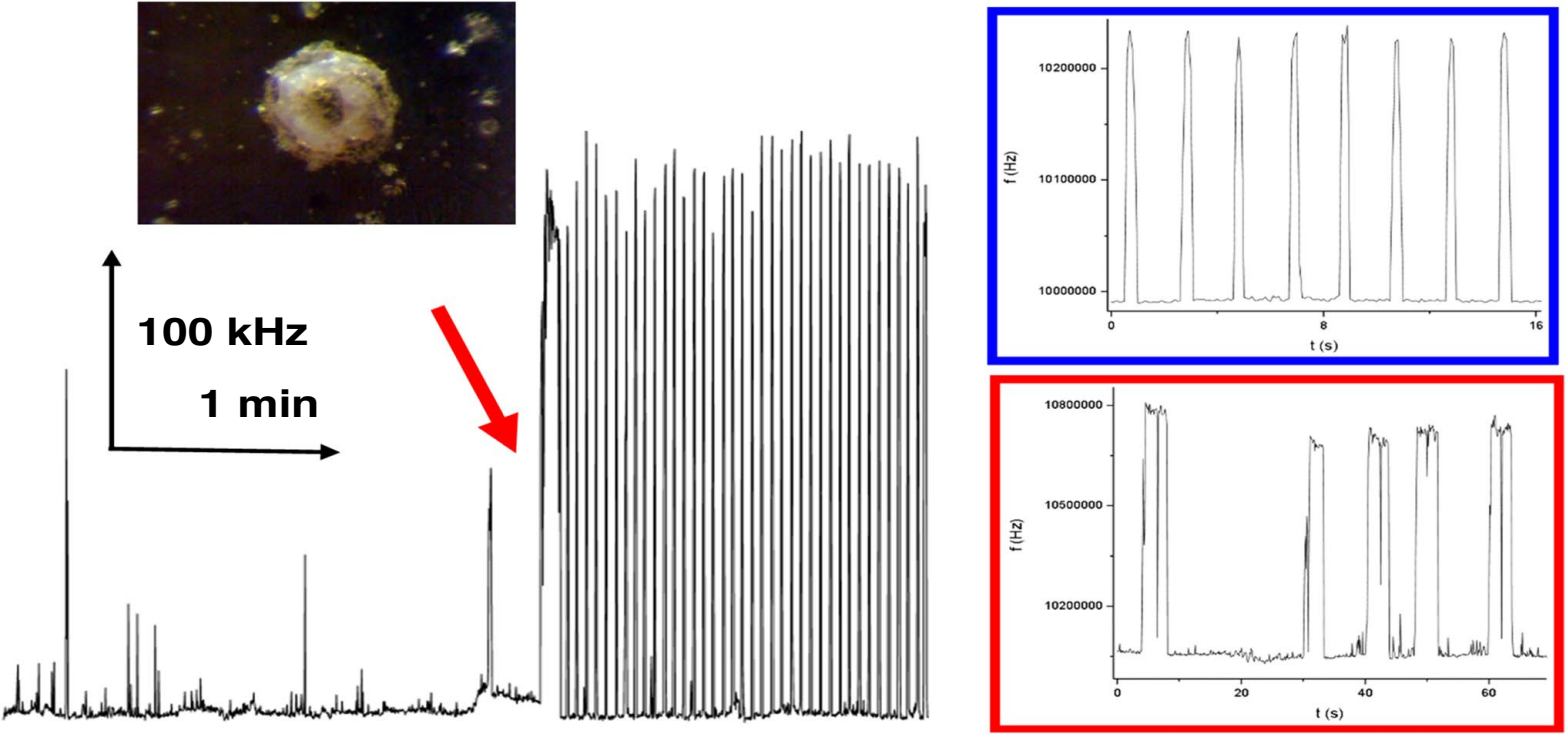
The cluster of cardiomyocyte cells (shown in the image) attached on the surface of 10 MHz PZ crystal modified with gelatin. The beating was started by the addition of 10 µmol L^−1^ isoproterenol (red arrow). Comparison of beating frequency peaks in the initial control state (blue rectangle) and after addition of 70 µmol L^−^.^1^ metoprolol as β-blocker (right). Data obtained from [61]

Eventually, an interesting approach focuses on mechanical events at the surface of PZ crystals. The cardiomyocytes (CM) cell lines provide the beating events typical for the heart. The confluent layer of CMs deposited on the PZ crystal surface was observed using fast-sampling impedance spectroscopy [59]. The CMs contractions were followed as periodic variations of the resonance frequency and bandwidth of the conductance peak; the resonance frequency changes were within some 25 Hz. A completely different situation was observed when CMs were deposited as a 3D cluster of cells—embryonic bodies; the whole bead-like cluster (Fig. 7, top left) will start beating synchronously and it functions as a “heart on chip” system. The beating and the associated forces can be followed using cantilevers known from atomic force microscopy (AFM) [60]; however, on the PZ surface, the beats are so strong that the resonator temporarily leaves the resonance region and extremely high frequency peaks appear (Fig. 7). The effects of drugs can be tested in real time; the right part of the figure presents signal traces in the presence of β-blocker—the beats are now different in shape—more chaotic, thought the size of peaks is up to 800 kHz compared to the control providing only 220 kHz.

The system allows to test the effects of different heart drugs; as the CMs can be obtained by differentiation of patient-derived stem cells, this might be a way towards personalized tuning of medication. From technical point of view, PZ crystal with oscillator is much more convenient transducer compared to AFM set-up.

## Piezosensors combined with other techniques

Electrochemical quartz crystal microbalance (EQCM) is the classic combination of electrochemistry and quartz crystal microbalance widely used since the 1980s [62]. The electrode of the PZ sensor becomes the working electrode of the electrochemical cell. The oscillator and potentiostat circuits should be properly separated.

Note that the amount of the electrogenerated species can be calculated from the passed charge using Faraday’s law and compared with the deposited mass obtained from the Sauerbrey’s Eq. (1); metal films are typically perfectly adhering and rigid.

PZ sensors are often considered as an alternative to optical surface plasmon resonance systems (SPR); interestingly, the gold PZ surface was modified with Au nanostructures to provide hybrid PZ/localized SPR sensing [63]. PZ sensors with Au electrodes were used together with surface-enhanced Raman spectroscopy (SERS) platform for detection of thrombin [64]. The functionalization process included a 1,6-hexanedithiol monolayer for covalent binding of Au NPs (20 nm); then, thiol-modified thrombin-specific aptamer was assembled. The presence of Au NPs amplified the frequency change significantly. The limit of detection for thrombin was 0.1 µM. Spectral ellipsometry carried out simultaneously with the QCM-D system allowed detailed characterization of polymer films [65]. The disaggregation reaction of single amyloid beta fibrils by anthocyanins was monitored using total-internal-reflection-fluorescence microscopy with a PZ sensor [66]. The disassembling of fibrils depends on the number of hydroxyl groups in the ring B of anthocyanin, and only delphinidin-3-galactoside (possessing three hydroxyl groups) showed high disassembly activity.

## Conclusions

In the 1990s, piezoelectric sensors were considered less sensitive than other transducers. However, as the theory for the function of piezosensors in liquids became more advanced and successful applications were continuously reported, this approach changed. For quick and easy detection of viruses, bacteria, proteins, nucleic acids, and small molecules such as drugs, hormones, and pesticides, piezoelectric biosensors are a suitable transducer. The mass changes traditionally considered for interpretation of response have been supplemented with dissipation and viscoelastic changes at the sensing surface, providing novel ways to perform sensitive assays. However, the exact response theories are not needed for bioanalytical use—the relationship between the measured signal and concentration of the analyte is simply obtained from the calibration curve; this works for mass changes, viscoelastic changes, and their combination as well.

Currently, piezosensors serve as useful tools for studying eukaryotic cells by providing real-time information on processes such as adhesion, changes in morphology, regulatory aspects, apoptosis, and allowing for testing of the physiological effects of new drugs, toxicity, and biocompatibility assays. Direct, label-free, real-time monitoring of affinity interactions with piezoelectric sensors is an economical alternative to other, often overpriced systems. The piezoelectric system is an open platform that allows moderately skilled researchers to build experimental devices from shell components. This results in a wide range of applications. Additionally, combining it with other sensing technologies can lead to innovative scientific achievements.

The aim of this contribution was to demonstrate different types of analytes measurable with piezosensors. In addition to the traditionally interpreted mass changes, additional sensing approaches appeared. On the other hand, piezosensors are also useful as a research tool for biochemistry and biology. The theoretical background for determination of kinetic rate and equilibrium constants was presented.

To conclude, the daily use of piezosensors often requires some active contribution from the users, which is stimulating for finding innovative ways, and promotes scientific progress.

## Data Availability

Not applicable.
